# Effect of tumour size ratio on liver recurrence-free survival of patients undergoing hepatic resection for colorectal liver metastases

**DOI:** 10.1186/s12885-022-09199-8

**Published:** 2022-01-25

**Authors:** Yana Zhai, Weijun Bai, Jin Zhou, Qian Dong, Jingdong Zhang

**Affiliations:** 1 Medical Oncology Department of Gastrointestinal Cancer, Liaoning Cancer Hospital & Institute, Cancer Hospital of China Medical University, No.44 Xiaoheyan Road, Dadong District, Liaoning Province, Shenyang, 110042 China; 2 Medical Oncology Department of Thoracic Cancer, Liaoning Cancer Hospital & Institute, Cancer Hospital of China Medical University, No.44 Xiaoheyan Road, Dadong District, Liaoning Province, Shenyang, 110042 China

**Keywords:** Colorectal liver metastases, Liver recurrence-free survival, Tumour size ratio, Tumour burden score

## Abstract

**Background:**

The study aimed to assess the impact of size differences of multiple liver metastases on liver recurrence-free survival (RFS) in patients undergoing hepatic resection for colorectal liver metastases (CRLMs).

**Methods:**

Overall, 147 patients with CRLMs who underwent hepatic resection between January 2010 and December 2016 were retrospectively analysed. Tumour size ratio (TSR) was defined as the maximum diameter of the largest liver lesion over the maximum diameter of the smallest liver lesion. The univariate and multivariate analyses were performed to determine independent prognostic risk factors. The prognostic value of the TSR was further explored in each Tumour Burden Score (TBS) zone. Log-rank survival analyses were used to compare liver RFS in the new clinical score and the Fong clinical score.

**Results:**

Based on the TSR, patients were classified into three groups: TSR < 2, 2 ≤ TSR < 4, and TSR ≥ 4. According to the multivariate analysis, TSR of 2–4 (hazard ratio [HR], 2.580; 95% confidence interval [CI] 1.543–4.312; P < 0.001) and TSR < 2 (HR, 4.435; 95% CI 2.499–7.872; P < 0.001) were associated with worse liver RFS. As TSR decreased, liver RFS worsened. TSR could further stratify patients in zones 1 and 2 into different risk groups according to the TBS criteria (zone 1: median liver RFS, 3.2 and 8.9 months for groups 1 and 2, respectively, *P* = 0.003; zone 2: median liver RFS, 3.5, 5.0, and 10.9 months for groups 1, 2, and 3, respectively, *P* < 0.05). The predictive ability of the new clinical score, which includes TSR, was superior to that of the Fong clinical score.

**Conclusions:**

TSR, as a prognostic tool, could accurately assess the effect of size differences across multiple liver metastases on liver RFS in patients undergoing hepatectomy for CRLMs.

**Trial registration:**

Retrospectively registered

## Background

Colorectal cancer (CRC) is the third most common cause of cancer-related deaths worldwide, with approximately 1.7 million new cases and 830,000 deaths each year [1, 2]. The most common site of distant metastases is the liver. More than half of the patients with CRC develop liver metastases, of which approximately 25% of patients present with simultaneous liver metastases, and 30%–40% of patients develop liver metastases during the course of the disease [3, 4]. Hepatic resection is the most effective curative-intent treatment for patients with colorectal liver metastases (CRLMs), with a five-year survival rate ranging from 40%–60% after surgery [5–8]. Although surgical treatment provides a potentially curable opportunity for patients with CRLMs, with significant improvement in the five-year survival, many patients relapse after hepatic resection. Approximately 40% of patients develop recurrence within 12 months after surgery [9]. Recurrence may affect patients’ long-term benefits from the surgery. Therefore, it is important to obtain clear prognostic information before hepatic resection.

Numerous studies have been conducted over the last 20 years for identifying clinicopathological, tumour morphological, and biological factors in patients with CRLMs as prognostic risk factors [10–15]. The prognostic risk factors determined by different research centres may vary, and the most consistent factors were used for establishing prognostic risk scores [16, 17]. The most widely used was the Fong clinical risk score [18], which was established in 1999 based on 1,001 patients with CRLMs who underwent hepatic resection. Based on the five risk factors in the Fong score, assigning each factor one point, the Fong score remains by far the most popular and extensively used clinical score because of its larger patient size and high quality. Several other clinical risk scores have also been established, notably, those reported by Zakaria et al. [17], Malik et al. [19], and Rees et al. [20]. These scoring systems usually include the number and size of liver metastases as prognostic risk factors. For instance, the Rees score considers > 3 hepatic metastases and a hepatic metastatic tumour measuring > 3 cm in diameter as poor prognostic factors. However, when multiple liver metastases occur, the predictive power of these factors alone is limited, and the effects of the size differences across multiple liver metastases on the prognosis of patients with CRLMs are rarely reported.

Therefore, this study aimed to assess whether size differences across multiple liver metastases affected liver recurrence-free survival (RFS) following curative-intent resection for CRLMs and assessed the effect of the tumour size ratio (TSR), i.e., maximum diameters of the largest and smallest liver lesions, on liver RFS.

## Methods

### Study design

Patients with CRLMs who underwent curative-intent liver resection at a single centre in China between January 1, 2010, and December 31, 2016, were consecutively included in the study. Patients who underwent ablation only, palliative liver resection (R2 resection), died perioperatively, had incomplete materials, or underwent combined hepatectomy and ablation for the tumour of maximum diameter were excluded. Patients who received adjuvant chemotherapy before surgery were also excluded. Among patients with extrahepatic disease during surgery, only patients who achieved an R0 resection of the extrahepatic disease were included in the study. The study was approved by the institutional review board of our hospital (No: 20200572); the requirement of informed consent was waived because of its retrospective nature. Detailed demographic and clinicopathologic data, including sex, age, primary tumour characteristics, preoperative carcinoembryonic antigen (CEA) levels, CRLM characteristics, operative details, and date of liver recurrence, were collected for each patient.

The primary tumour characteristics, including the tumour location (colon vs. rectum), American Joint Committee on Cancer primary tumour T stage and N stage, and tumour differentiation, were recorded.

CRLM characteristics such as the size and number of tumours, and the presence of unilobar or bilobar disease in the liver was also recorded. Data on operative details included the extent of liver resection (major vs. minor) and combined use of ablation. Major hepatectomy was defined as a resection of ≥ 3 Couinaud liver segments [21].

Preoperative assessment included thoracic, abdomen, and pelvis computed tomography (CT) and magnetic resonance imaging (MRI) of the liver. Synchronous liver metastases were defined as metastases detected within three months of diagnosis of primary colorectal cancer. The primary CRC characteristics and the number and size of liver metastases were determined using final histopathological reports.

The maximum diameters of the largest and smallest liver lesions were recorded, and their ratio was defined as the TSR. As Sasaki et al. previously proposed, Tumour Burden Score (TBS) was defined on a Cartesian plane that incorporated both maximum tumour size and lesion number, which was calculated as TBS^2^ = (maximum tumour diameter)^2^ + (number of liver lesions)^2 ^[22]. The Fong clinical score were as follows: primary tumour lymph node positive (1 point), interval between primary resection and diagnosis of liver metastases of < 12 months (1 point), number of CRLM > 1 (1 point), preoperative CEA level of > 200 ng/mL (1 point), and maximum CRLM diameter > 5 cm (1 point) [18]. Follow-up data were obtained from the hospital’s information department.

### Definition of end points and related indicators

The primary endpoint of this study was liver RFS. The liver RFS for each patient was calculated as the time interval from the date of the liver surgery to the date of liver recurrence or the last follow-up. CT and MRI were used for evaluating liver recurrence, and the evaluation date was used as the recurrence confirmation date. In patients without liver recurrence, the latest imaging evaluation date was used as the censored date. Intraoperative ultrasound was performed for confirming the findings of preoperative imaging and for assisting in surgical planning.

### Statistical analyses

To assess the impact of TSR, the cut-off values of TSR were selected to divide the patients into three groups: group 1, lowest 25% of TSR; group 2, between the 25^th^–90^th^ percentile of TSR; and group 3, the highest 10% of TSR [23]. The demographic and clinicopathological characteristics of the study population were stratified according to different TSRs. The summary statistics were presented as total counts and frequencies for the categorical variables and median values with interquartile ranges (IQRs) for the continuous variables. The differences between the three groups were assessed using the chi-squared test and Fisher's exact test as appropriate. Liver RFS was estimated using the Kaplan–Meier method, and the differences in the liver RFS were assessed with the log-rank test.

The Cox proportional hazards regression model was used for identifying the independent predictors of liver RFS in the multivariate analysis, and the results were reported as hazard ratios (HRs) with 95% confidence intervals (95% CIs). The factors included in the multivariate model were selected based on clinical relevance and statistical significance according to the univariate analysis (*P* ˂0.05).

To compare the prognostic ability of the new prognostic model (including TSR) and the Fong clinical score, the area under the receiver operating characteristic curve (AUC) was used with the outcome of interest being liver RFS.

A two-tailed P-value of < 0.05 was considered statistically significant. All analyses were performed using SPSS software version 20.0 (IBM SPSS, Chicago, IL, USA).

## Results

### Baseline characteristics of the patients

A total of 147 patients who underwent hepatic resection for CRLMs at our hospital were included in the study, with a median follow-up of 36.6 (IQR, 9.2–41.3) months.

According to the cumulative percentage of TSR distribution, the optimal cut-off points for the TSR were 2 (28%, lowest 25%) and 4 (88%, highest 10%). The demographic and clinicopathological characteristics of the entire cohort stratified using TSR are presented in Table 1.

**Table 1:** Baseline demographic and clinicopathological characteristics stratified using the tumour size ratio

Variables	Number of Patients	Tumour Size Ratio			*P*-value
		< 2 (*n* = 41)	≥ 2– < 4 (*n* = 70)	≥ 4 (*n* = 36)	
Age (years)					
≤ 60	80 (54.4)	20 (48.8)	40 (57.1)	20 (55.6)	0.686
˃60	67 (45.6)	21 (51.2)	30 (42.9)	16 (44.4)	
Sex					
male	90 (61.2)	26 (63.4)	42 (60.0)	22 (61.1)	0.938
female	57 (38.8)	15 (36.6)	28 (40.0)	14 (38.9)	
Primary Tumour Location					
Colon	69 (46.9)	23 (56.1)	28 (40.0)	18 (50.0)	0.238
Rectum	78 (53.1)	18 (43.9)	42 (60.0)	18 (50.0)	
T category					
T1–T3	72 (49.0)	18 (43.9)	30 (43.5)	24 (66.7)	0.067
T4	72 (49.0)	21 (51.2)	39 (55.7)	12 (33.3)	
Missing	3 (2.0)	2 (4.9)	1 (1.4)	-	
N category					
N0 + N1	107 (72.8)	28 (68.3)	46 (65.7)	33 (91.7)	0.019
N2	37 (25.2)	11 (26.8)	23 (32.9)	3 (8.3)	
Missing	3 (2.0)	2 (4.9)	1 (1.4)	-	
Primary Tumour Differentiation					
Well + Moderate	90 (61.2)	29 (70.7)	38 (54.3)	23 (63.9)	0.219
Poor + Others	47 (32.0)	9 (22.0)	26 (37.1)	12 (33.3)	
Missing	10 (6.8)	3 (7.3)	6 (8.6)	1 (2.8)	
Preoperative CEA (ng/mL)					
≤ 200	124 (84.4)	33 (80.5)	63 (90.0)	28 (77.8)	0.142
˃200	21 (14.3)	7 (17.1)	6 (8.6)	8 (22.2)	
Missing	2 (1.4)	1 (2.4)	1 (1.4)	-	
Extrahepatic Disease					
No	120 (81.6)	35 (85.4)	58 (82.9)	27 (75.0)	0.471
Yes	27 (18.4)	6 (14.6)	12 (17.1)	9 (25.0)	
Presentation					
Synchronous CRLM	98 (66.7)	27 (65.9)	50 (71.4)	21 (58.3)	0.396
Metachronous CRLM	49 (33.3)	14 (34.1)	20 (28.6)	15 (41.7)	
Size of the Largest CRLM, Median (IQR)	3.0 (2.0–4.0)	3.0 (1.5–4.2)	2.5 (1.5–3.5)	4.0 (2.8–6.0)	0.001
Number of CRLMs, Median (IQR)	3.0 (2.0–5.0)	2.0 (2.0–3.0)	3.0 (2.0–5.0)	4.0 (3.0–5.7)	0.005
Location on the Liver					
Unilobar Disease	54 (36.7)	20 (48.8)	22 (31.4)	12 (33.3)	0.166
Bilobar Disease	93 (63.3)	21 (51.2)	48 (68.6)	24 (66.7)	
Type of Surgical Procedure					
Resection	109 (74.1)	35 (85.4)	49 (70.0)	25 (69.4)	0.154
Resection + Ablation	38 (25.9)	6 (14.6)	21 (30.0)	11 (30.6)	

Overall, the median age of patients was 59.0 years (IQR, 54.0–64.0 years), and most patients were men (*n* = 90, 61.2%). Primary lesions were found in the colon in 69 patients (46.9%) and in the rectum in 78 patients (53.1%). Based on the surgical records and final histopathological reports, 72 patients (49.0%) had T4 primary tumours, and 37 patients (25.2%) had a primary node status of ≥ 4. The median preoperative CEA level was 18.9 ng/mL (IQR, 5.0–90.0 ng/mL).

Several demographic and clinicopathological characteristics were similar between the three groups (all *P* > 0.05). For example, the primary tumour location was comparable between the three groups (*P* = 0.290). However, a primary node status of ≥ 4 was less frequent in group 3 than in the other groups (*P* = 0.019).

Regarding the extent of liver disease, the number of liver metastases in all patients was ≥ 2, with an average of 3 (IQR, 2–5). The median size of the largest metastatic liver lesion was 3.0 cm (IQR, 2.0–4.0 cm). Most patients had synchronous CRLM presentation (*n* = 98, 66.7%). Regarding the type of liver resection, 95 patients (64.6%) underwent minor hepatectomy, while 52 patients (35.4%) underwent major hepatectomy. There were 27 patients with M1 extrahepatic disease in the cohort. The distribution of extrahepatic metastases was as follows: the diaphragm (*n* = 10), abdominal wall (*n* = 15), and unilateral adrenal (*n* = 2). These patients underwent a complete extrahepatic R0 resection.

### Univariate and multivariate analyses of liver RFS

In the univariate analysis, the factors found to be significantly associated with a shorter liver RFS were an advanced primary tumour stage (T4), a tumour lymph node status of ≥ 4, a CEA level of ≥ 200 ng/mL, a TSR of 2–4, and a TSR of < 2. In the multivariate analysis, after the model was adjusted for all significant factors, a CEA level of ≥ 200 ng/mL (HR, 2.765; 95% CI 1.540–4.840; *P* < 0.001), a TSR of 2–4 (HR, 2.580; 95% CI 1.543–4.312; *P* < 0.001), and TSR of < 2 (HR, 4.435; 95% CI 2.499–7.872; *P* < 0.001) were all associated with a poor liver RFS. The details of the univariate and multivariate analyses of the liver RFS in the overall cohort are summarized in Table 2.

**Table 2:** Univariate and multivariate Cox regression analyses of the liver recurrence-free survival in the overall cohort

	**Univariate Analysis**		**Multivariate Analysis**	
	**HR (95% CI)**	***P*-value**	**HR (95% CI)**	***P*-value**
Age (years)				
≤ 60	1.000 (reference)			
> 60	1.263 (0.877–1.819)	0.210		
Sex				
Male	1.000 (reference)			
Female	1.036 (0.711–1.509)	0.854		
Primary Tumour Location				
Colon	1.000 (reference)			
Rectum	0.905 (0.628–1.304)	0.593		
Primary T Stage				
T1–T3	1.000 (reference)		1.000 (reference)	
T4	1.707 (1.164–2.504)	0.006	1.429 (0.953–2.143)	0.084
Primary N Stage				
N0 + N1	1.000 (reference)		1.000 (reference)	
N2	1.763 (1.156–2.690)	0.008	1.313 (0.846–2.038)	0.225
Primary Tumour Differentiation				
Well + Moderate	1.000 (reference)			
Poor + Others	0.750 (0.501–1.122)	0.162		
Preoperative CEA (ng/mL)				
≤ 200	1.000 (reference)		1.000 (reference)	
> 200	2.010 (1.204–3.354)	0.008	2.765 (1.540–4.840)	< 0.001
Extrahepatic Disease				
No	1.000 (reference)			
Yes	1.034 (0.649–1.646)	0.888		
Tumour Distribution				
Unilobar	1.000 (reference)			
Bilobar	0.819 (0.565–1.188)	0.293		
Tumour Burden Score				
< 3	1.000 (reference)			
≥ 3– < 9	1.487 (0.580–3.817)	0.409		
≥ 9	1.585 (0.688–3.654)	0.279		
Presentation				
Synchronous	1.000 (reference)			
Metachronous	0.760 (0.512–1.127)	0.172		
Extent of liver resection				
Minor	1.000 (reference)			
Major	0.822 (0.560–1.206)	0.316		
Type of Resection				
Resection	1.000 (reference)			
Resection + Ablation	1.013 (0.673–1.525)	0.950		
Tumour size ratio				
≥ 4	1.000 (reference)		1.000 (reference)	
≥ 2– < 4	2.368 (1.457–3.848)	< 0.001	2.580 (1.543–4.312)	< 0.001
< 2	4.442 (2.582–7.644)	< 0.001	4.435 (2.499–7.872)	< 0.001

### Prognostic significance of TSR in each TBS zone

**Fig. 1:**
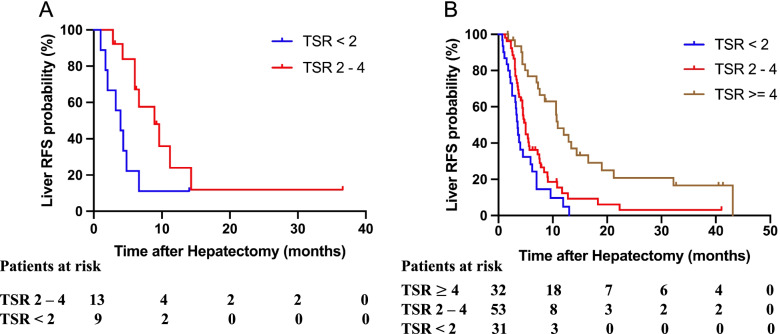
Kaplan–Meier analysis of the liver RFS for different TSR groups in zones 1 and 2, **A** different TSR groups in zone 1, **B** different TSR groups in zone 2

TBS [22] is a predictive indicator that captures the cumulative impact of tumour size and number on prognosis. It is a useful tool for assessing the impact of tumour morphology on prognosis in patients with CRLMs undergoing hepatic resection. To further explore the function of TSR, TBS was introduced, and the prognostic effects of TSR in each TBS zone were assessed. A prognostic risk model using the TBS cut-off values was developed as follows: TBS < 3 (zone 1), 9 > TBS ≥ 3 (zone 2), and TBS ≥ 9 (zone 3). Patients were divided into three zones according to the above criteria [zone 1: *n* = 22 (15.0%); zone 2: *n* = 116 (78.9%); and zone 3: *n* = 9 (6.1%)]. In zone 1, there were 22 patients including 9 patients (40.9%) in group 1, 13 (59.1%) in group 2, and no patients in group 3. The median liver RFS values were 3.9 and 8.9 months for groups 1 and 2, respectively. The Kaplan–Meier analysis showed significant differences in the liver RFS between the two groups (log-rank, *P* = 0.031) (Fig. 1a).

In zone 2, a total of 116 patients were included, with 31 patients (26.7%) in group 1, 53 (45.7%) in group 2, and 32 (27.6%) in group 3. The median liver RFS values were 3.5, 5.0, and 10.9 months for groups 1, 2, and 3, respectively. TSR was used to group the patients into three risk groups with significantly different liver RFS outcomes. Specifically, an incremental worsening of liver RFS was noted as TSR decreased (log-rank test: group 1 vs. group 2, *P* = 0.045; group 1 vs. group 3, *P* < 0.001; and group 2 vs. group 3, *P* < 0.001) (Fig. 1b). In zone 3, there were nine patients, including one patient (11.1%) in group 1, four (44.4%) in group 2, and four (44.4%) in group 3. The Kaplan–Meier analysis showed that there were no significant differences in the liver RFS across the groups.

### Prognostic significance of TSR in a new clinical score

The Fong clinical score has been the most widely used clinical score to date [18]. Among the five prognostic risk factors, there are two factors related to the liver. We hypothesised that the TSR had a better predictive ability than the two factors, and a new clinical score was established by replacing the above two factors with TSR. The new clinical score was composed of the following factors: (1) primary tumour lymph node positive (1 point), (2) preoperative CEA level of > 200 ng/mL (1 point), (3) interval between primary resection and diagnosis of liver metastases of < 12 months (1 point), and (4) TSR (0 points assigned for a TSR ≥ 4, 1 point assigned for 4 > TSR ≥ 2, and 2 points assigned for a TSR < 2).

According to the new clinical score, patients were divided into low-risk (score 0**–**1), medium-risk (score 2**–**3), and high-risk groups (score ≥ 4). For comparison, patients were also divided into different risk groups according to the Fong score as described earlier [24]: low-risk (score 0**–**1), medium-risk (score 2**–**3), and high-risk groups (score ≥ 4).

**Fig. 2:**
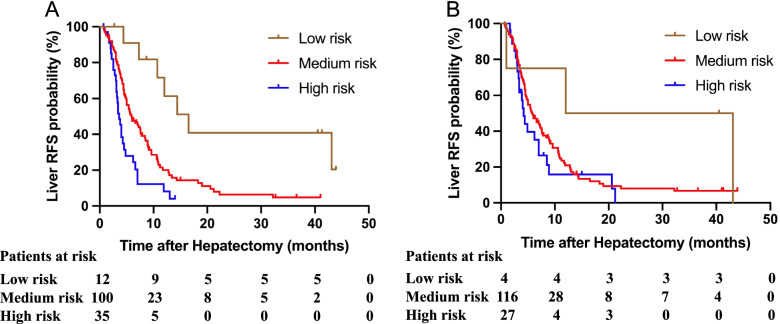
Kaplan–Meier analysis of the liver RFS stratified using the new score and the Fong score, **A** by the new score risk groups, **B** by the Fong score risk groups

Liver RFS was compared between the three risk groups using Kaplan–Meier analysis. In the new clinical score, the low-risk group had a better liver RFS than the medium-risk group (median liver RFS, 16.5 vs. 5.9 months; *P* = 0.002), and the medium-risk group had a better liver RFS than the high-risk group (median liver RFS, 5.9 vs. 3.6 months; *P* = 0.002) (Fig. 2a). In the Fong clinical score, the low-risk group had a higher liver RFS than the medium-risk group (median liver RFS, 12.0 vs. 5.7 months; *P* = 0.120), and the medium-risk group had a higher liver RFS than the high-risk group (median liver RFS, 5.7 vs. 4.2 months; *P* = 0.234); however, no significant differences were detected across the three groups (Fig. 2b).

The discriminatory ability of the two clinical scores was assessed. The predictive ability of the new clinical score was superior to that of the Fong clinical score (AUC of 0.659 vs. 0.570, respectively).

## Discussion

Hepatic resection is the main treatment for patients with CRLMs, supported by a reasonable theoretical basis and a large amount of clinical data [25]. Neoadjuvant chemotherapy (NC) has also been applied to increase the resectability rate, although it has not been associated with a difference in overall survival [26]. Numerous risk scores have been developed, and various clinicopathological and biological factors have been used to group patients with CRLMs into distinct prognostic categories [10–12, 18, 27–29] over the last two decades. For example, the Fong score proved to be a sufficient independent prognostic factor, and a Fong score of three or higher was found to be an independent risk factor associated with decreased survival [26, 30]. The factors associated with the liver usually included the tumour size and number. An increased tumour size and several metastases were considered negative predictors of the long-term survival. Additionally, the number and size of liver lesions were evaluated dichotomously, such as whether the tumour number was > 1 or whether the tumour size was > 5 cm [12–14, 18, 20]. In our study, we confirmed that in patients with CRLMs, the extent of the liver lesions was an independent predictor after hepatic resection. Moreover, our study developed a new prognostic factor, TSR. We found that TSR remained strongly associated with liver RFS. Specifically, a decrease in TSR was strongly associated with an incrementally worse liver RFS. After adjusting for other competing risk factors, patients with a TSR of < 2 had almost a 4.5-fold higher risk of liver recurrence following the resection of CRLMs.

Recently, some studies showed that TBS was a tool that could accurately estimate the impact of tumour morphology on long-term survival in patients undergoing resection of CRLMs [22]. The recently developed genetic and morphological evaluation score [27] also identified TBS as one of the prognostic factors. TBS could stratify patients with CRLMs into different prognostic groups with respect to the long-term prognosis. Particularly, gradual worsening of long-term survival was observed as TBS increased in our study. Notably, patients with CRLM were stratified into significantly different risk groups according to TSR in TBS zones 1 and 2 but not in zone 3; when TBS is < 9, TSR can further predict the prognosis of patients with CRLMs within the same TBS zone. The larger the size difference in multiple liver metastases, the longer the liver RFS and better the prognosis of patients with CRLM. These results highlight the prognostic role of TSR, which could be used as a new risk stratification tool in addition to TBS for patients with CRLMs.

To further explore the prognostic effect of TSR, a new clinical score with TSR was developed in our study. The three risk groups assigned using the new score were used for stratifying patients into distinct prognostic groups. In contrast, the grouping of patients according to the Fong score resulted in extremely poor prognostic discrimination. This result is consistent with that in previous studies [17, 28, 31] that have challenged the prognostic power of the existing clinical risk scores. The outstanding performance of the new score might be associated with TSR. The prognostic superiority is based on the composition of TSR, as it could fully reflect the size differences across multiple liver lesions; thus, the extent of disease could be reflected completely. In contrast, a simple classification of tumour morphological characteristics involving the optimal cut-off value will inevitably result in the loss of prognostic information. Additionally, when multiple liver metastases exist, a single cut-off value often fails to account for the size differences across multiple tumours. For example, when all the multiple tumours were < 5 cm, there were no risk factors according to the Fong score, whereas based on our TSR prognostic model, the risk groups were determined by the size differences across multiple lesions.

This study had several limitations that should be considered while interpreting the findings. First, the study was retrospective, and selection bias was largely unavoidable. The function of TSR should be further confirmed in a prospective study with a larger number of patients with CRLM following hepatic resection. Second, for the purpose of analysis, TSR was calculated using data from pathological specimens and not from preoperative radiographic images. Although the results have proven that the size and number of liver metastases based on the resected specimens are highly correlated with preoperative imaging findings [32], additional studies are needed for further confirmation so that risk assessments could be made before surgery. Last, the prognostic value of TSR was established among a cohort of patients from a single institution, and external validation should be conducted in the future.

## Conclusions

In summary, our study suggested that TSR may play an important role in assessing the prognosis of patients with CRLM undergoing radical resection of multiple liver metastases. The larger the size differences across multiple liver metastases, the longer the liver RFS of patients with CRLM after surgery. Further, when TBS is < 9, TSR might be an effective supplement to TBS for predicting prognoses of CRLMs.

## Data Availability

The datasets generated and/or analysed during the current study are not publicly available due to confidential patient data but are available from the corresponding author on reasonable request.

## Abbreviations

AUC: Area under the receiver operating characteristic curve
CEA: Carcinoembryonic antigen
CIs: Confidence intervals
CRC: Colorectal cancer
CRLMs: Colorectal liver metastases
CT: Computed tomography
HRs: Hazard ratios
IQRs: Interquartile ranges
MRI: Magnetic resonance imaging
NC: Neoadjuvant chemotherapy
RFS: Recurrence-free survival
TBS: Tumour burden score
TSR: Tumour size ratio
